# Supplementary material for: The Essential and the Nonessential Roles of Four Clock Elements in the Circadian Rhythm of Metarhizium robertsii
Source: J Fungi (Basel). 2022 May 25;8(6):558. doi: 10.3390/jof8060558 (PMC9224670; doi:10.3390/jof8060558)
Supplement: Supplementary file 1 [file jof-08-00558-s001.zip › jof-1738251-supplementary.pdf]

**Table S1.** Paired primers used for manipulation of target genes in *M. robertsii*.

| **Primers** | **Paired sequences (5′–3′)*** | **Purpose** |
|---|---|---|
| cFrh -F/R | CAATCACAAACACCTTCAAAATGGATGAGTTGTTTGATGTTTTT / TCCTCGCCCTTGCTCACCATAAGGTACAAGCTCTGCGCAG | Cloning *frh* cDNA (3294 bp) for fusion to *gfp* |
| cFrq -F/R | CAATCACAAACACCTTCAAAATGCCATTGCAGAATCGAA / TCCTCGCCCTTGCTCACCATACTCTCTTCATCGCTGGTGC | Cloning *frq* cDNA (3018 bp) for fusion to *gfp* |
| upFrh-F/R | TGGACGAGCTGTACAAGTAAAAGGCGTTTCGTATTGAGCA / AAGCTTGGCTGCAGGTCGACCTCGCATCTATGGACGGTTT | Cloning *frh* 5′-end (1461 bp) for recombination /deletion |
| dnFrh-F/R | ACGTCGACCCATGGCTCGAGCCAAACGCCTTCGCATAA / CGTTAACACTAGTCAGATCTTCATCATCATCCAGCACCAG | Cloning *frh* 3′-end (1382 bp) for recombination /deletion |
| upFrq -F/R | TGGACGAGCTGTACAAGTAAACCTATTATTACGACGAGATTGAG / AAGCTTGGCTGCAGGTCGACTGAAAGCGGAGGCTACATAC | Cloning *frq* 5′-end (1384 bp) for recombination /deletion |
| dnFrq -F/R | ACGTCGACCCATGGCTCGAGTACGGCGTCAGAATACCAGT / CGTTAACACTAGTCAGATCTGCAATCAAAGAATCGCAAAG | Cloning *frq* 3′-end (1584 bp) for recombination /deletion |
| flFrh-F/R | ATCCGTCGACCTGCAGCC**AAGCTT**CAGCCTTATTCATTACTTTGGA / ACACTAGTCAGATCTTC**TAGTGT**ATGGCGTATTGGGTTTATTT | Cloning full-length *frh* (6734 bp) for complementation |
| flFrq-F/R | ATCCGTCGACCTGCAGCC**AAGCTT**CAAACGGAGCCGCAAAATA / ACACTAGTCAGATCTTC*T***AGTGT**TCGCCCGCTGTCAAGAAT | Cloning full-length *frq* (4975 bp) for complementation |
| pFrh -F/R | GATGCGAAGCGGATGGAT / GGCACCACCAGAACCTTGAT | PCR detecting *frh* |
| pFrq -F/R | TGCTTGACCTCCCATCTCTC / TTTCTTGGTAAAGTAGACTGGTATTC | PCR detecting *frq* |
| qFrh -F/R | TGGATTGCCAAGATTCATCA / CTTCTTGTTCTTGCCCTTGC | qPCR detecting *frh* |
| qFrq -F/R | TCGCCAGGAAACTCGGTAAC / AACGTTGCAGTCGGGTTTTG | qPCR detecting *frq* |
| qWC1-F/R | AAGAGCTCATGCGAAAGGAA / GAGCATCACCCGGATACAGT | qPCR detecting *wc1* |
| qWC-F/R | CGAAGACGTCGCTATGATGA / ATGTGCTTGCACTTCCACTG | qPCR detecting *wc2* |
| q18S-F/R | GAGCCAGCGAGTAATTCC / AGCCATTCAATCGGTAGTAG | qPCR detecting 18 sRNA |

*The underlined regions are the fragments for the fusion of *frh or frq* to *gfp*, the homogeneous recombination for targeted gene deletion or the gateway exchange for targeted gene complementation at the enzyme sites of *Hind*III/*Xba*I in bold red.

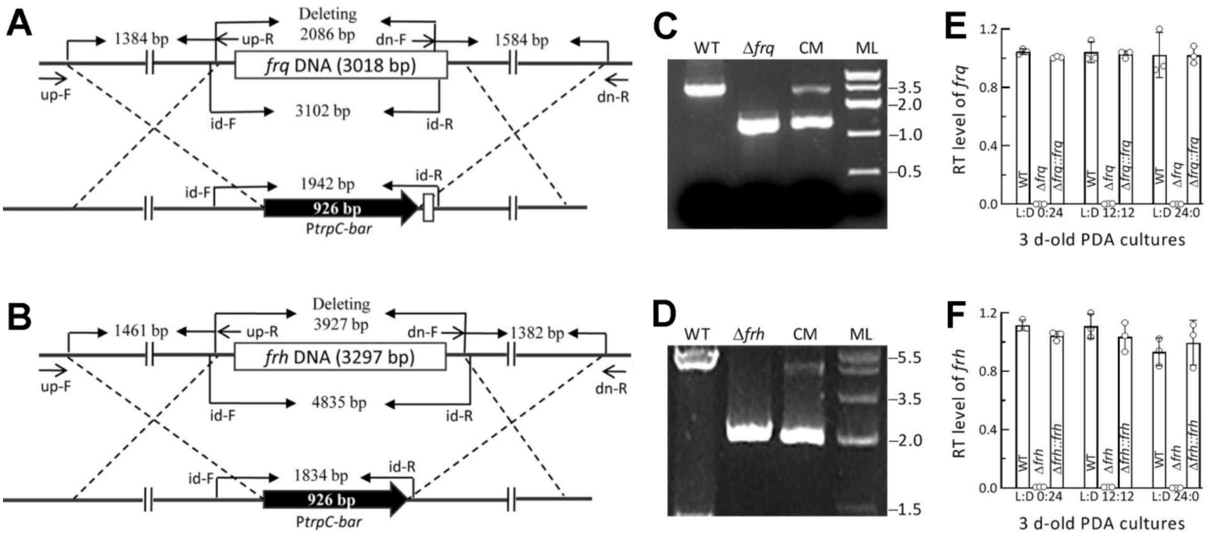


**Figure S1.** Generation and identification of *frq* and *frh* mutants in *M. robertsii*. (**A, B**) Diagrams for the deletion strategies of *frq* and *frh*. (**C, D**) The *frq* and *frh* mutants identified via PCR analysis. Indicated by the detected DNA fragments, the coding and partial flanking regions of each target gene were successfully deleted from the WT strain as expected (3102 + 926 – 1942 = 2086 bp for *frq*; 4835 + 926 – 1834 = 3927 bp for *frh*). CM, complementation mutant. ML, molecular ladder of genomic DNA. (**E, F**) Relative transcript (RT) levels of *frq* and *frh* in the 3 d-old PDA cultures of their mutants versus the WT strain at 25°C in indicated L:D cycles. Error bars: SDs from three cDNA samples derived from independent cultures.
